# Regulating Zn Deposition via an Artificial Solid–Electrolyte Interface with Aligned Dipoles for Long Life Zn Anode

**DOI:** 10.1007/s40820-021-00599-2

**Published:** 2021-02-23

**Authors:** Kai Wu, Jin Yi, Xiaoyu Liu, Yang Sun, Jin Cui, Yihua Xie, Yuyu Liu, Yongyao Xia, Jiujun Zhang

**Affiliations:** 1 Institute for Sustainable Energy/College of Sciences, Shanghai University, 99 Shangda Road, Shanghai, 200444 People’s Republic of China; 2 School of Materials, Sun Yat-Sen University, Guangzhou, 510006 People’s Republic of China; 3 Department of Chemistry and Institute of New Energy, Fudan University, Shanghai, 200433 People’s Republic of China

**Keywords:** Regulated Zn deposition, Artificial solid–electrolyte interface, Perovskite type dielectric material, Zn anode, Zn ion battery

## Abstract

**Highlights:**

- An artificial solid–electrolyte interface composed of a perovskite type material, BaTiO_3_, is introduced to Zn anode surface in aqueous zinc ion batteries.
- The BaTiO_3_ layer endowing inherent character of the switched polarization can regulate the interfacial electric field at anode/electrolyte interface.
- Zn dendrite can be restrained, and Zn metal batteries based on BaTiO_3_ layer show stable cycling.

**Abstract:**

Aqueous zinc ion batteries show prospects for next-generation renewable energy storage devices. However, the practical applications have been limited by the issues derived from Zn anode. As one of serious problems, Zn dendrite growth caused from the uncontrollable Zn deposition is unfavorable. Herein, with the aim to regulate Zn deposition, an artificial solid–electrolyte interface is subtly engineered with a perovskite type material, BaTiO_3_, which can be polarized, and its polarization could be switched under the external electric field. Resulting from the aligned dipole in BaTiO_3_ layer, zinc ions could move in order during cycling process. Regulated Zn migration at the anode/electrolyte interface contributes to the even Zn stripping/plating and confined Zn dendrite growth. As a result, the reversible Zn plating/stripping processes for over 2000 h have been achieved at 1 mA cm^−2^ with capacity of 1 mAh cm^−2^. Furthermore, this anode endowing the electric dipoles shows enhanced cycling stability for aqueous Zn-MnO_2_ batteries. The battery can deliver nearly 100% Coulombic efficiency at 2 A g^−1^ after 300 cycles.
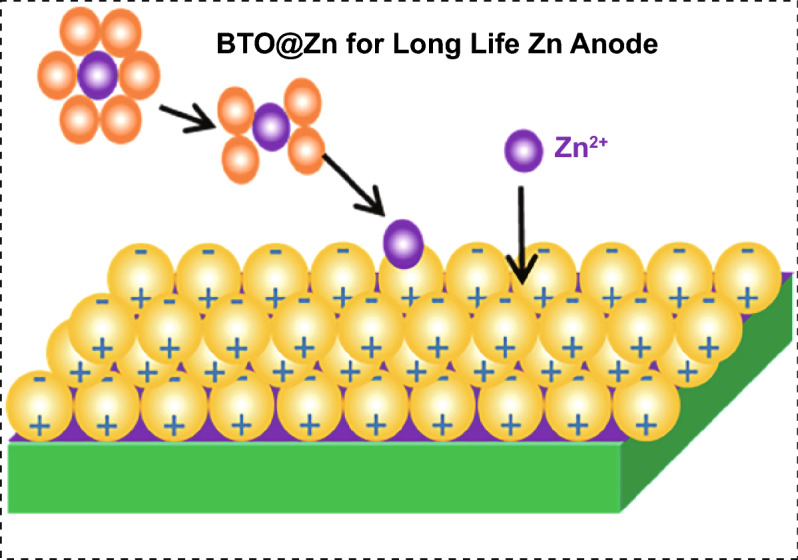

**Supplementary Information:**

The online version of this article (10.1007/s40820-021-00599-2).

## Introduction

Aqueous zinc ion battery (ZIB) is considered to be a competitive alternative for next-generation renewable energy storage devices by virtue of high theoretical volume capacity of metal Zn anode (5854 mAh cm^−3^), nontoxicity and high safety [1–4]. By now, several rechargeable ZIBs employing mild acid aqueous electrolyte, such as Zn-MnO_2_ [5–7], Zn-V_2_O_5_ [8–11], Zn-hexacyanoferrate [12, 13] and so on, have delivered favorable electrochemical performance. However, the challenge related to Zn dendrite growth is still an urgent issue, which impacts the future practical application.

**Fig. 1:**
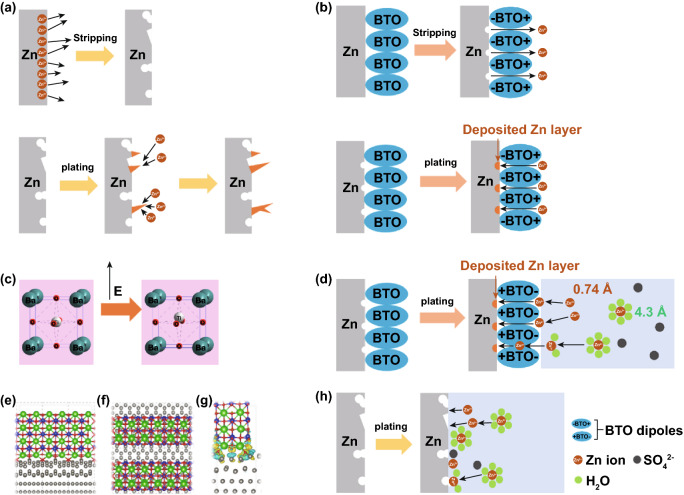
Schematic of zinc ion transport during Zn stripping/plating for: **a** bare Zn, **b** BTO@Zn foil. **c** Schematic diagram of the Ti ion migration in the [TiO_6_] octahedral interstitial sites under the external electric field. **d** Schematic of the mechanism of zinc ion transport at the BTO@Zn/electrolyte interface during Zn plating process. Schematic illustration of the BTO@Zn surface used in the simulation: **e** side view, **f** top view. The bottom two Zn layers are fixed during the geometrical optimization. Ba, Ti, O, Zn atoms are represented by green, blue, red, gray spheres, respectively. **g** Differential charge density (*ρ*_diff_ = *ρ*_BTO@Zn_ – *ρ*_BTO_ – *ρ*_Zn_) of the BTO@Zn surface. Yellow and blue surfaces indicate the electron gain and loss, respectively. **h** Schematic diagram of zinc ion transport at the Zn anode/electrolyte interface during Zn plating process

Unfortunately, compared to nonaqueous lithium-ion batteries, there is lack of a solid–electrolyte interface (SEI) in aqueous ZIBs, which could effectively protect Zn anode [14–17]. Accordingly, an artificial SEI introduced to the surface of Zn anode would be a promising strategy to suppress zinc dendrites. CaCO_3_ [18], polyamide [19], TiO_2_ [20, 21], metal–organic framework [22, 23], ZnO [24], hydrogen-substituted graphdiyne [25], etc., serving as artificial SEI layers, have successfully facilitated uniform Zn plating/stripping. Furthermore, it would show positive prospects that an artificial SEI controlling electric field at the interface would promote even Zn plating/stripping. Guided by the controlled interfacial electric field, homogenous charge would distribute at the interface, availing the redox kinetic processes in battery, where reversible process of even Zn plating/stripping could be effortless [26]. Subsequently, Zn dendrite would be confined. Whereupon, how to achieve the ability to control the interfacial electric field for the artificial SEI is significant. As is well known, dielectric materials can be polarized under an external electric filed [27]. In addition, the polarization can be parallel to the applied electric field. BaTiO_3_ (BTO), a typical perovskite type material with ABO_3_ structure, has been currently employed for energy storage composite [28]. Under the applied electric field, the polarization of an ionically bonded crystal dielectric material is produced, which results from the movement of cation from the center of the symmetry site [29]. In BTO structure, Ti^4+^ cation is in the [TiO_6_] octahedral interstitial sites [30]. Thus, under an external electric field, Ti^4+^ could be deviated from the center of the [TiO_6_]. Subsequently, electric dipoles induce polarized electric field on the surface. Moreover, the polarization can be switched by the external electric field [31–33]. As a proof-of-concept, BTO is captured to construct the artificial SEI, producing directional electric field to regulate Zn solvation/deposition. Figure 1 illustrates the proposed process of zinc ion migration on bare Zn and BTO-coated Zn foil (BTO@Zn) during Zn plating/stripping. Zinc ion can be stripped in order from BTO@Zn, while the unordered zinc ion movement takes place on the bare zinc foil surface during stripping process in Fig. 1a, b. It is speculated that both the uniform ion pathways and directional dipoles supplied by the BTO layer can guide ordered migration of zinc ions. The ion pathways physically restrain the disorderly movement of Zn^2+^, and dipoles construct even electric field at SEI to make zinc ions liberate in order. Additionally, zinc ions can be regulated to electroplate uniformly, resulting from the directional dipoles switched by the applied electric field. A dense deposited Zn layer without dendrites is formed between BTO layer and Zn anode (Fig. 1b). On the contrary, Zn prefers to nucleate locally, then accumulates at the initial deposited area on bare Zn foil during plating process. Hence, irregular zinc dendrites are formed. Compared to bare Zn foil, Zn dendrite growth would be restrained effectively by BTO layer. Favorable electrochemical performance has been obtained in the symmetric cells and aqueous ZIBs based on BTO@Zn foil. The uniform Zn plating in the symmetric cells with BTO@Zn has been achieved at the current density of 1 mA cm^−2^ with areal capacity of 1 mAh cm^−2^ over 1000 cycles. When the current density and areal capacity are raised up to 5 mA cm^−2^ and 2.5 mAh cm^−2^, respectively, the symmetric cell with BTO@Zn can operate over 1500 cycles. Impressively, compared to the ones with bare Zn, aqueous Zn-MnO_2_ batteries employing BTO@Zn anode reveal enhanced electrochemical performances and deliver nearly 100% Coulombic efficiency at 2 A g^−1^ after 300 cycles.

## Experimental

### Materials

BaTiO_3_ (BTO, < 100 nm) and ZnSO_4_·7H_2_O were purchased from Aladdin, China. KMnO_4_ was purchased from Energy Chemistry, China. Ti foil was 0.03 mm.

### Preparation of BTO@Zn Foil

A slurry was composed of BTO and poly(vinylidene fluoride) (PVDF) in a weight ratio of 9:1 in *N*-methyl pyrrolidone (NMP) solvent, then which was coated on Zn foil. The foil was dried in vacuum at 80 °C overnight. The areal loading of BTO was about 1 mg cm^−2^.

### Preparation of MnO_2_ Electrode

KMnO_4_ (2 g) powders were calcined at 500 °C for 5 h at the condition of 5 °C min^−1^, then naturally cooled down to room temperature. Afterward, the obtained powders were washed by centrifugation with distilled water and ethanol until the purple of solution was eliminated. The products were dried at 80 °C for 12 h. The cathode was mixed by 70 wt% of as-prepared MnO_2_, 20 wt% Super P and 10 wt% poly(tetrafluoroethylene) (PTFE) to form thin film. After drying, the thin film was pressed at Ti mesh. The areal loading of MnO_2_ was about 2 mg cm^−2^.

### Material Characterization

The morphologies were detected by using a scanning electron microscope (SEM, HITACHI S-4800). XRD patterns of the samples were obtained using powder XRD analysis (Bruker D8 Advance, Germany) with Cu Kα radiation (*λ* = 0.15406 nm), operating at 40 kV × 40 mA.

### Electrochemical Tests

All of the cells and batteries were assembled as coin cells (CR2016) in ambient environment. In the symmetric cells, Zn (thickness ~ 0.03 mm, a disk of 12 mm in diameter) or BTO@Zn and glass fiber served as electrode and separator, respectively. The Zn–Ti cells employed Ti foil as the working electrode and zinc or BTO@Zn foil worked as counter and reference electrodes. To investigate the nucleation behavior, the cutoff voltage was set as 1 V at the current density of 5 mA cm^−2^ with the areal capacity of 5 mAh cm^−2^. Tafel plot measurement was carried out at 5 mV s^−1^ with Zn or BTO@Zn as the working electrode, Pt foil as the counter electrode and Ag/AgCl as the reference electrode. The electrolyte for the symmetric cells was 2 M ZnSO_4_ aqueous solution prepared by ZnSO_4_⋅7H_2_O and distilled water. To verify the feasibility of BTO@Zn, Zn–MnO_2_ batteries based on bare Zn and BTO@Zn were fabricated, respectively. Zn or BTO@Zn was used as the anode, MnO_2_ was the cathode and glass fiber was the separator. The electrolyte for Zn–MnO_2_ batteries was 2 M ZnSO_4_ and 0.1 M MnSO_4_ aqueous solution. The cyclic voltammogram (CV) profiles were carried out on an electrochemical workstation CHI760E (ChenHua, Shanghai, China) at a scan rate of 1 mV s^−1^ under potential control (1–1.8 V). MnO_2_ served as a working electrode and zinc foil worked as counter and reference electrodes. Electrochemical impedance spectroscopy (EIS) was conducted on a Solartron analytical electrochemical workstation (1470E) in the frequency range of 10^–2^–10^6^ Hz. Galvanostatic charge/discharge cycling measurements were conducted on a LANHE CT2001A (Wuhan, China) battery testing instrument.

### Calculations

Density functional theory calculations (DFT) were conducted utilizing the Vienna Ab initio Simulation Package (VASP) within the projector augmented-wave approach [34–36]. Generalized gradient approximation (GGA) in the parameterization of Perdew, Burke, and Ernzerhof (PBE) pseudopotential was utilized to confirm the exchange–correlation potential [37]. The dimension of the supercell is 8.0 × 13.8 × 27.4 Å^3^, containing 72 Zn atoms, 16 Ba atoms, 18 Ti atoms and 54 O atoms. The plane-wave cutoff was controlled to 520 eV. Geometry optimizations were carried out by taking advantage of a conjugate gradient minimization until all of the forces acting on ions were less than 0.01 eV/Å per atom. The Γ point was used in for the Brillouin zone sampling. The formation energy of depositing a Zn atom on the bare Zn or BTO@Zn surface is evaluated by the following equations:

1 $$ E_{{{\text{bare}}\;{\text{Zn}}}}^{f} = E_{{{\text{Zn\_atom@bare\_Zn}}}} - E_{{{\text{Zn\_atom}}}} - E_{{{\text{bare\_Zn}}}} $$

2 $$ E_{{{\text{BTO@Zn}}{.}}}^{f} = E_{{{\text{Zn\_atom@BTO@Zn}}}} - E_{{{\text{Zn\_atom}}}} - E_{{\text{BTO@Zn}}} $$

where the $E_{{{\text{Zn\_atom}}}}$ is the energy of a single Zn atom; $E_{{{\text{bare\_Zn}}}}$ and $E_{{\text{BTO@Zn}}}$ are the energy of bare Zn surface and BTO@Zn surface, respectively; $E_{{{\text{Zn\_atom@bare\_Zn}}}}$ is the total energy of bare Zn surface with an extra Zn atom; $E_{{{\text{Zn\_atom@BTO@Zn}}}}$ is the total energy of BTO@Zn surface with an extra Zn atom.

## Results and Discussion

### Mechanism of Zn^2+^ Transport at Anode/Electrolyte Interface

To further understand the evolution of Zn stripping/plating, a detailed depiction is also shown in Fig. 1. Under an external electric field, the Ti ion can be deviated from the center of the symmetrical site, [TiO_6_] (Fig. 1c). It is considered that BTO can be polarized by the external field during charging/discharging processes [38]. Thereby, a directional electric field on BTO layer surface is provided when discharging. Moreover, the BTO layer renders considerably uniform ion pathways. Hence, zinc ions can be stripped from anode in order, subsequently enter into the electrolyte. These zinc ions coordinate with solvents generating the hydrated Zn ions (Zn(H_2_O)_6_^2+^) until the stripping process terminates. Subsequently, during plating process, Zn(H_2_O)_6_^2+^ ions are required to undergo desolvation process during which Zn(H_2_O)_6_^2+^ ions (4.3 Å) transform into Zn^2+^ ions (0.74 Å) [39]. In the following, zinc ions are plated on the Zn anode surface. However, for the bare Zn, only a few nuclei sites induce zinc ion to nucleate, which would cause the uneven Zn plating [40]. In addition, during the desolvation process of Zn(H_2_O)_6_^2+^ ion, the species of decomposition, passivation and by-product would be produced and adhere to the Zn anode surface [41]. Fortunately, in Fig. 1d, the direction of BTO polarization is switched by the employed electric field during plating process. Negative charges will concentrate on the element O of Ti–O due to the surface ordered electric field of BTO. A certain amount of SO_4_^2−^ anions could be rejected by the BTO layer due to the electrostatic repulsion. The interaction between cations and anions gets weak; then, the side reactions would be suppressed [42]. Additionally, the water molecules of hydrated zinc ion can be attracted by the element O of Ti–O, which enriches negative charges. It is possible that hydrogen bond can be constructed between the H atom of H_2_O and the O atom of Ti–O. A solid–liquid interface could be formed to promote the fast ion transfer [43]. Herein, benefiting from the ordered electric field, the free de-hydrated zinc ions could be accelerated to pass through BTO layer. More nucleation sites can be provided for even Zn plating. Furthermore, density functional theory (DFT) calculations were performed to demonstrate the merit of the BaTiO_3_ absorbate in electrochemically depositing Zn metal. In Fig. 1e, f, a BTO nano-rod is put on top of the Zn layer to simulate the BTO@Zn surface. A charge transfer from the Zn atoms to the O atoms of BTO was identified (Fig. 1g). Compared with the bare Zn surface, the formation energy of depositing an extra Zn atom on the BTO@Zn surface is much lower (− 2.28 vs. − 0.35 eV), suggesting that the electrochemical Zn plating would preferentially take place in the vicinity of the BTO area. Therefore, it could be reasonably concluded that the electrochemical Zn deposition could be regulated by the presence of BTO adsorbates. In addition, the ion pathways furnished by BTO layer physically restrict two-dimension diffusion of zinc ions at the interface on account of the BTO electrochemical inertia [44]. Fortunately, the BTO SEI facilitates the dense Zn plating without dendrites. On the contrary, certain hydrated Zn^2+^ ions and sulfate would be attracted in the vicinity of bare Zn anode. After performing the desolvation process, zinc ions migration would be obstructed by these ions; subsequently, Zn would be gradually plated at the local area (Fig. 1h). The Zn dendrites are inevitably produced.

### Electrochemical Properties of the Symmetric Cells

**Fig. 2:**
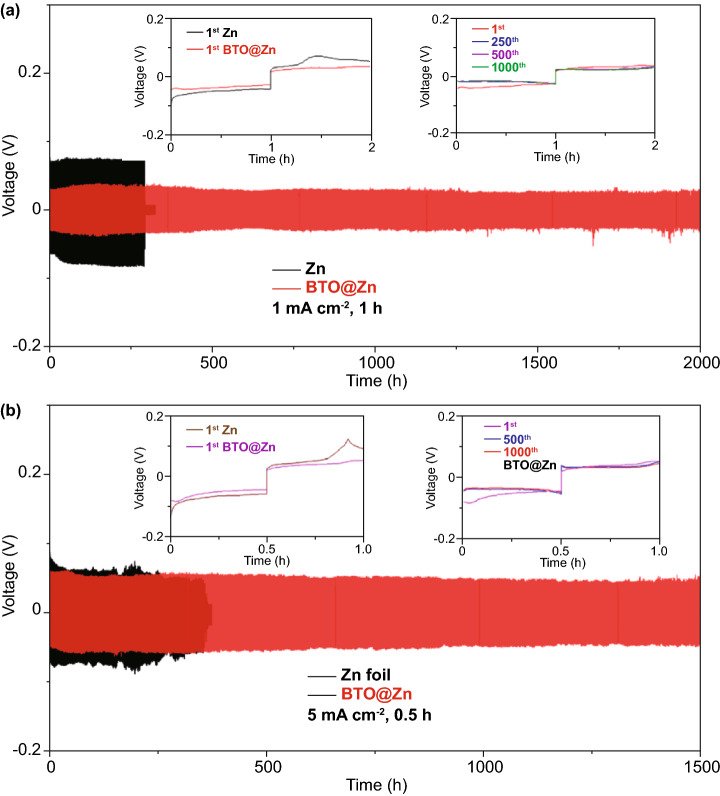
Cycling performance of the symmetric cells with Zn and BTO@Zn, respectively: **a** at 1 mA cm^−2^ with capacity of 1 mAh cm^−2^ and **b** at 5 mA cm^−2^ with capacity of 2.5 mAh cm^−2^. The insets reveal the detailed corresponding voltage profiles at various current densities and different cycles

Compared to the XRD patterns of commercial BTO and Zn foil, it can be found that BTO is successfully coated on Zn foil, as demonstrated by the typical diffraction peaks are observed at 22.1° and 31.5° (Fig. S1). The size of BTO is less than 100 nm, determined by scanning electron microscopy (SEM) image (Fig. S2). An artificial SEI has been constructed on Zn foil through coating BTO layer (~ 1 μm) (Fig. S3). Generally, Zn suffers from corrosion in the electrolyte. Under the protection of BTO layer, the Zn anode corrodes less, which is further demonstrated by Tafel plot in Fig. S4. The corrosion potential of BTO@Zn shows no obvious change in comparison with the one of bare Zn, while it can be found that lower corrosion current is achieved for BTO@Zn. The anti-corrosion property would enhance the electrochemical performance of BTO@Zn. To further evaluate the behavior of Zn plating/stripping, galvanostatic cycling measurements are performed. As displayed in Fig. 2, a tip can be obviously observed in the voltage profiles of the initial charge process in the symmetric cell based on bare Zn (Zn-symmetric cell), which could be ascribed to the spatial variation of Zn dendrites along the bare Zn anode surface [45]. In contrast, the voltage profiles of the initial charge process in the symmetric cell based on BTO@Zn (BTO@Zn-symmetric cell) are smoother, whose polarization is lower than that of the Zn-symmetric cell. The ordered zinc ion migration can be obtained under the electric field, which is furnished by the directional dipoles of BTO layer. Therefore, the Zn dendrite growth is suppressed and the kinetics of zinc ion migration is enhanced. Subsequently, after cycling at 1 mA cm^−2^ with capacity of 1 mAh cm^−2^, the BTO@Zn-symmetric cell exhibits an improved cycling stability over 2000 h in Fig. 2a. Negligible difference of the 250th, 500th and 1000th cycles displays remarkable cycling stability. When the current density and areal capacity are increased to 5 mA cm^−2^ and 2.5 mAh cm^−2^, respectively, the BTO@Zn-symmetric cell operates for over 1500 h (Fig. 2b). The voltage profiles of the 500th and 1000th cycles are nearly in accordance. The rate performance of BTO@Zn-symmetric cell foil is illustrated in Fig. S5. At 8 mA cm^−2^ with capacity of 8 mAh cm^−2^, the voltage profiles of BTO@Zn-symmetric cell keep stable while the one employing bare Zn foil fails. In order to explore the positive properties of the BTO interphase for the symmetric cell, the electrochemical impedance technique was employed. Compared with the Zn-symmetric cell, it can be found that the reduced charge transfer resistance is observed for BTO@Zn-symmetric cell (Fig. S6), which can be attributed to the even zinc ion migration on the artificial interphase. The even zinc ion migration can be achieved under the electric filed, which is provided by the aligned dipoles of BTO. Therefore, the reversible Zn plating/stripping is enhanced and Zn dendrites are alleviated. It is reasonably concluded that the BTO layer enables Zn anode to achieve long-term cycle stability and high rate performance. In Fig. S7a, the polarization voltage of BTO@Zn is 84 mV in the BTO@Zn–Ti cell, which is smaller than that of bare Zn foil (115 mV) in the Zn–Ti cell. The Coulombic efficiency (CE) of bare Zn foil is 89%, which is lower than that of BTO@Zn (97.8%) for the first cycle in Fig. S7b. The higher Coulombic efficiency is obtained in the BTO@Zn–Ti cell due to the reduced side reactions caused by the SO_4_^2−^ anions. A certain amount of SO_4_^2−^ anions could be repelled by the BTO layer, which restricts the interaction between cations and anions. Accordingly, the side reactions would be restrained. Moreover, the desolvation process of hydrated zinc ion possibly benefits from the attraction of the O with enriched negative charge. Hydrogen bond perhaps forms to promote the H_2_O removal from hydrated Zn^2+^. Therefore, Zn plating needs less energy. It is noteworthy that the nucleation stage is vital for understanding behaviors of Zn dendrites [46]. There is a voltage dip at the beginning of the Zn plating, which indicates Zn nucleates on the surface of electrodes. The voltage dip for BTO@Zn is smaller than that of bare Zn. The nucleation overpotential of BTO@Zn is 39 mV in comparison with 49 mV of Zn, clarifying that BTO layer could facilitate Zn ion transport due to the dipoles. Additionally, at this impetuous current condition, the Zn–Ti cell holds for only 8 cycles (16 h), but the BTO@Zn–Ti cell can operate for 14 cycles (28 h) (Fig. S7b). It is indicated that the reversibility of Zn plating/stripping could be enhanced by the BTO SEI.

**Fig. 3:**
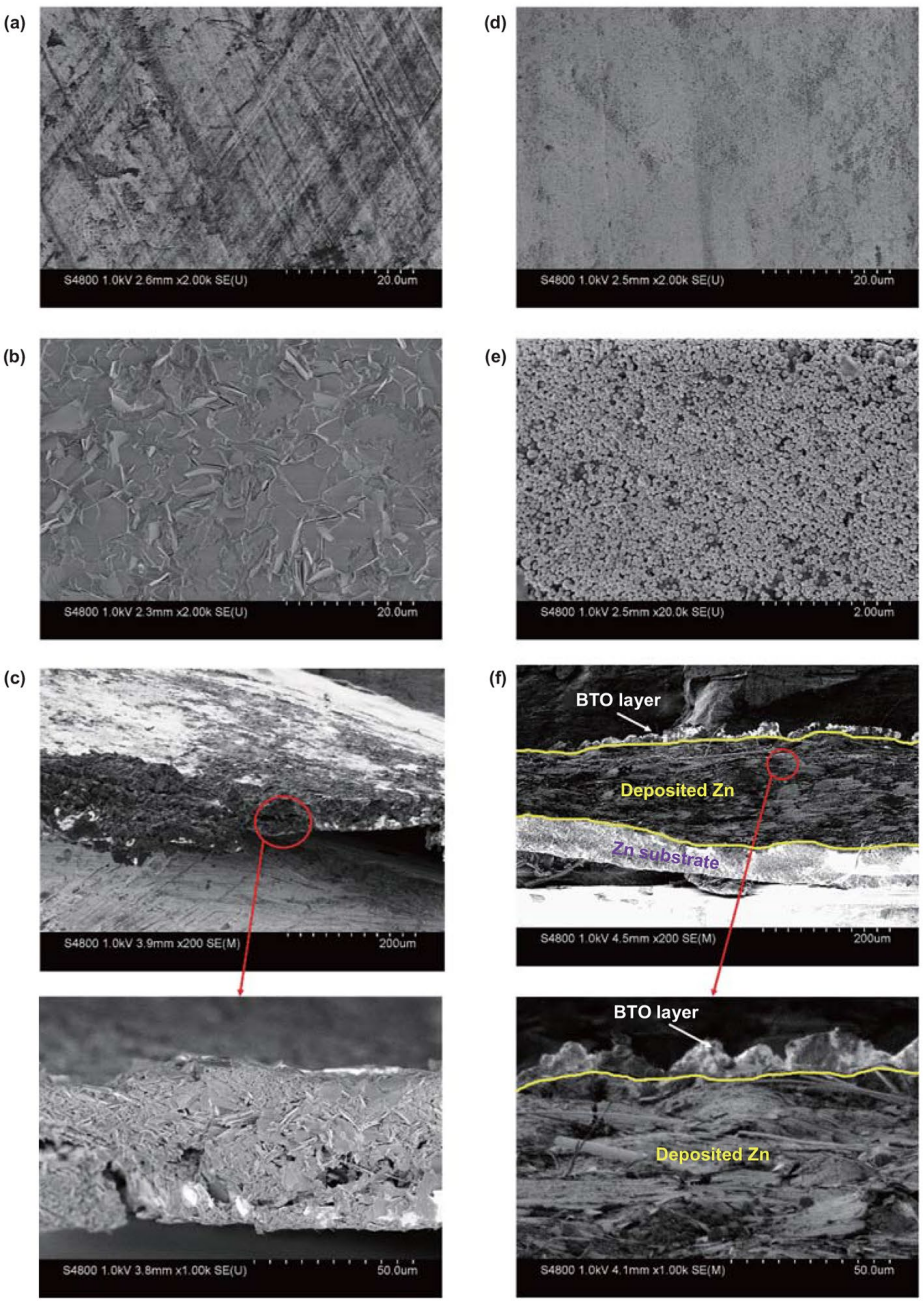
SEM images of the anode morphology: **a–c** bare Zn and **d–f** BTO@Zn foils. **a**, **d** before and **b**, **c** and **e**, **f** after 100 cycles at 1 mA cm^−2^ and 1 mAh cm^−2^ of the symmetric cells. Highlight typical cross section SEM images in **c** and **f**. BTO layer, deposited Zn and Zn foils are separated by the yellow lines. (Color figure online)

To further understand the process of Zn plating/stripping, the morphologies of various Zn electrodes are displayed by SEM images in Fig. 3. Figure 3a presents the surface of bare Zn foil, before cycling. After 100 cycles at 1 mA cm^−2^ with capacity of 1 mAh cm^−2^, Zn is plated unevenly on bare Zn foil, which can be obviously observed in Fig. 3b. It is noteworthy that Zn is liable to be plated at the initial nucleation area, and then, protrusions grow up. Moreover, bare Zn is severely pulverized and the morphology changes impressively (Fig. 3c). By contrast, the morphology of BTO@Zn could remain smooth and flat after cycling (Fig. 3d before and Fig. 3e after cycling). Compared to the case in Fig. 3c, a dense uniform Zn layer is plated between BTO layer and Zn foil in the enlarged SEM image of Fig. 3f. As it is well known, the by-product Zn_4_SO_4_(OH)_6_∙5H_2_O would be produced on the bare Zn anode during cycling, which could affect the Zn deposition negatively. Although it can be found that the by-product is produced on BTO layer as well (Fig. S8), the side reaction could be reduced to a certain degree, which is consistent with the higher Coulombic efficiency of BTO@Zn–Ti cells. Under the same experimental conditions, the BTO-protected Zn delivers higher reversible Zn plating/stripping processes. Moreover, it also shows a typical diffraction peak at 31.5° in Fig. S8, which demonstrates the high stability of BTO after long cycling.

### Electrochemical Performance of the Zn-MnO_2_ Batteries

**Fig. 4:**
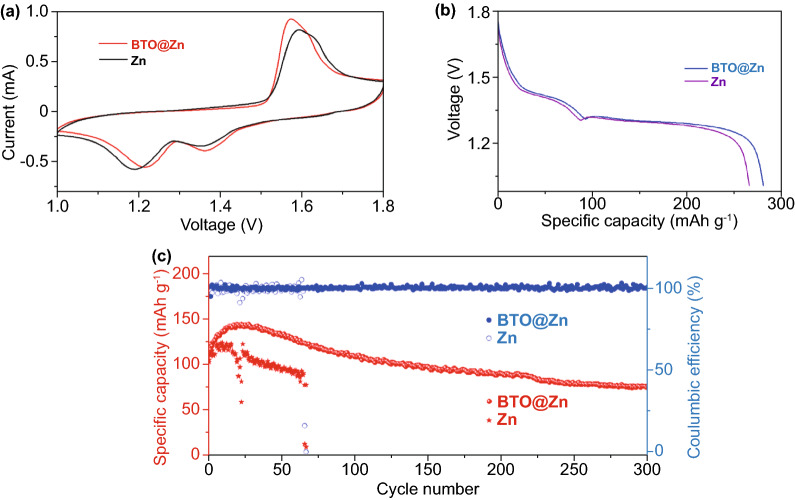
Electrochemical performance of Zn–MnO_2_ batteries based on bare Zn and BTO@Zn foil, respectively. **a** CV profiles of the 2nd cycle at 1 mV s^−1^. **b** GCD curves of the 2nd discharge process at 200 mA g^−1^. **c** Cycling performance at 2 A g^−1^

To clarify the feasibility of this potential strategy for practical application, Zn–MnO_2_ batteries using bare Zn and BTO@Zn (BTO@Zn–MnO_2_ batteries) are fabricated and tested. XRD pattern of the material cathode reveals monoclinic structure (Fig. S9). The SEM image exhibits flake-like morphology of MnO_2_ in Fig. S10. 0.1 M MnSO_4_ served as additive to alleviate the dissolution of Mn^2+^ derived from the reduction of Mn^4+^ [47]. The cyclic voltammograms (CV) of both batteries are presented in Fig. 4a. The cathodic peaks of BTO@Zn–MnO_2_ battery at 1.22/1.36 V are higher than the ones (1.19/1.35 V) of Zn–MnO_2_ battery based on bare Zn, indicating the higher kinetics of zinc ion insertion in BTO@Zn–MnO_2_ battery. Additionally, to further demonstrate the fast kinetics of zinc ion insertion in BTO@Zn–MnO_2_ batteries, the discharge galvanostatic intermittent titration technique (GITT) is carried out. As presented in Fig. S11, a higher discharge plateau is observed in the BTO@Zn–MnO_2_ battery, which indicates the faster kinetics of zinc ion insertion. The favorable kinetics of zinc ion insertion in BTO@Zn–MnO_2_ battery can be ascribed to uniform ion pathways and the regulated zinc ion migration on the BTO@Zn surface. Similarly, benefitting from the BTO layer, the fast kinetics of Zn plating can be achieved during the charging process. The surface electric field of BTO is switchable along with the applied electric field. Uniform Zn plating can be achieved under the even electric field at the anode/electrolyte interface. It can be found that the anodic peak for BTO@Zn foil at 1.57 V is smaller than the one (1.59 V) for bare Zn, which could be ascribed to the kinetics of Zn plating caused by the uniform zinc ion migration. Coincident conclusion can be obtained in galvanostatic charge/discharge (GCD) curves for the two batteries. In Fig. 4b, two discharge plateaus are observed in the Zn–MnO_2_ batteries. During discharging process, the manganese oxidation state is changed from Mn(IV) to Mn(III)/Mn(II), which are corresponding to the formation of Zn_*x*_MnO_2_ and ZnMn_2_O_4_ [48]. The BTO@Zn–MnO_2_ battery shows a higher discharge plateau compared to the Zn–MnO_2_ battery, which is corresponded to the CV profiles. The rate capabilities of Zn–MnO_2_ batteries based on Zn and BTO@Zn are shown in Fig. S12. It can be found that the BTO@Zn–MnO_2_ battery shows higher rate capability, which further demonstrates the faster kinetics of Zn ion migration on BTO@Zn due to the BTO layer. Impressively, at 2 A g^−1^, the battery with BTO@Zn anode displays a capacity retention of 67% after 300 cycles; however, the battery with Zn anode reveals only 16% capacity retention at the 66th cycle and fails at the next cycle (Fig. 4c). It manifests BTO@Zn can undergo fierce stripping/plating to ensure nearly 100% Coulombic efficiency after 300 cycles at 2 A g^−1^.

## Conclusion

Zn dendrite growth is one of the serious problems affecting the stability of Zn anode. Engineering an artificial SEI on the surface of Zn is an effective strategy to restrain Zn dendrite growth. In this work, it is demonstrated that the artificial SEI based on BTO could effectively suppress Zn dendrites. The BTO layer provides numerous aligned dipoles whose electric polarization can be switched with the applied electric field. During cycling process, even electric field on the surface of BTO layer can induce the ordered zinc ion migration. Compared to bare Zn, BTO@Zn anode possesses remarkable cycling stability and rate performance. The BTO@Zn-symmetric cell presents a decent cycling stability over 2000 h (1000 cycles) at 1 mA cm^−2^ with capacity of 1 mAh cm^−2^. What is more, when the current density and areal capacity is 5 mA cm^−2^ and 2.5 mAh cm^−2^, respectively, the BTO@Zn-symmetric cell can operate over 1500 cycles. Demonstrated in aqueous Zn–MnO_2_ batteries, the BTO@Zn–MnO_2_ battery reveals a higher rate capability and nearly 100% Coulombic efficiency after 300 cycles at 2 A g^−1^. This study is expected to enlighten the further research in Zn anode.

## Supplementary Information

Below is the link to the Supplementary Information.

Supplementary file 1 (PDF 564 kb)
