# Supplementary material for: Regulating Zn Deposition via an Artificial Solid–Electrolyte Interface with Aligned Dipoles for Long Life Zn Anode
Source: Nanomicro Lett. 2021 Feb 23;13:79. doi: 10.1007/s40820-021-00599-2 (PMC8187518; doi:10.1007/s40820-021-00599-2)
Supplement: Supplementary file 1 — (PDF 564 kb) [file 40820_2021_599_MOESM1_ESM.pdf]

Nano-Micro Letters

Supporting Information for

Regulating Zn Deposition via an Artificial Solid-Electrolyte Interface with Aligned Dipoles for Long Life Zn Anode

Kai Wu,[1] Jin Yi,[1*] Xiaoyu Liu,[1] Yang Sun,[2*] Jin Cui,[1] Yihua Xie,[3] Yuyu Liu,[1] Yongyao Xia,[3] and Jiujun Zhang[1*]

[1]Institute for Sustainable Energy/College of Science, Shanghai University, 99 Shangda Road, Shanghai, 200444, P. R. China

[2]School of Materials, Sun Yat-sen University, Guangzhou 510006, P. R. China

[3]Department of Chemistry and Institute of New Energy, Fudan University, Shanghai 200433, P. R. China

* Corresponding author. E-mail: jin.yi@shu.edu.cn (Jin Yi); sunyang5@mail.sysu.edu.cn (Yang Sun); jiujun.zhang@i.shu.edu.cn (Jiujun Zhang)

Supplementary Figures

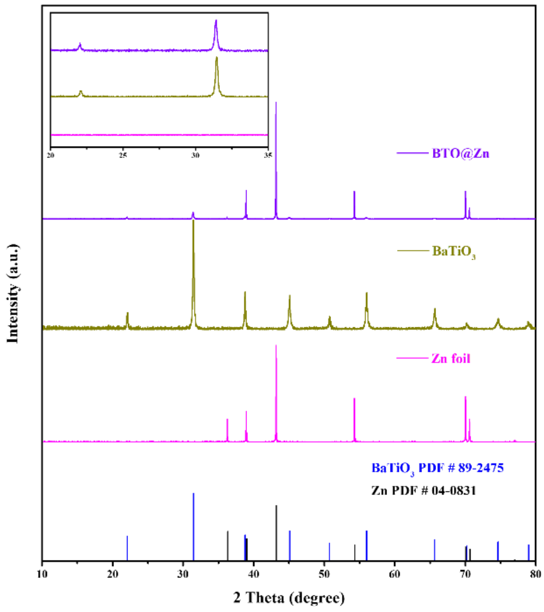

Fig. S1 XRD patterns of Zn foil, BTO and BTO@Zn

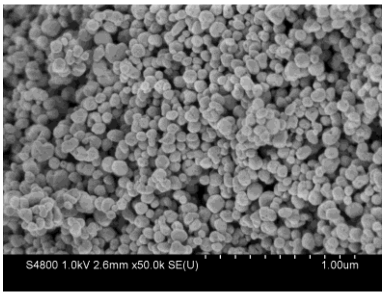

Fig. S2 SEM image of BTO nano particles

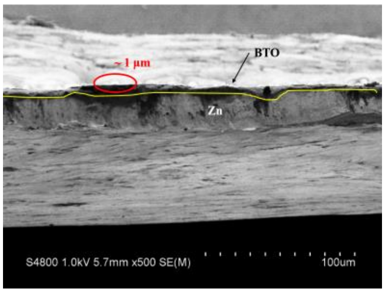

Fig. S3 Cross-section SEM image of BTO@Zn

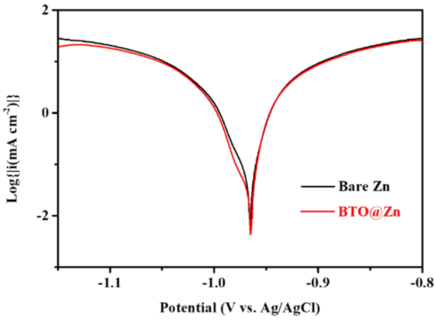

Fig. S4 Tafel plot measurements for the corrosion of bare Zn and BTO@Zn

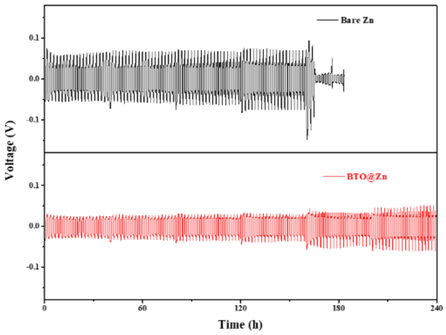

Fig. S5 Voltage profiles of the Zn-symmetric cells with bare Zn and BTO@Zn at 0.2, 0.5, 1, 2, 5 and 8 mA cm^{-2} for every 20 cycles

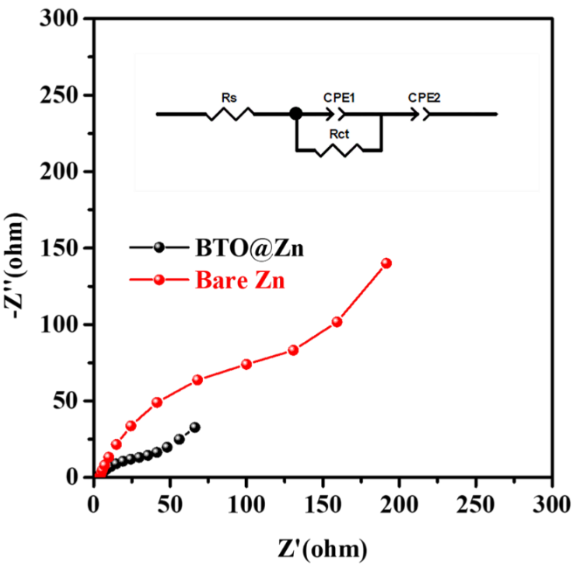

Fig. S6 Nyquist plots of the pristine BTO@Zn-symmetric and Zn-symmetric cells

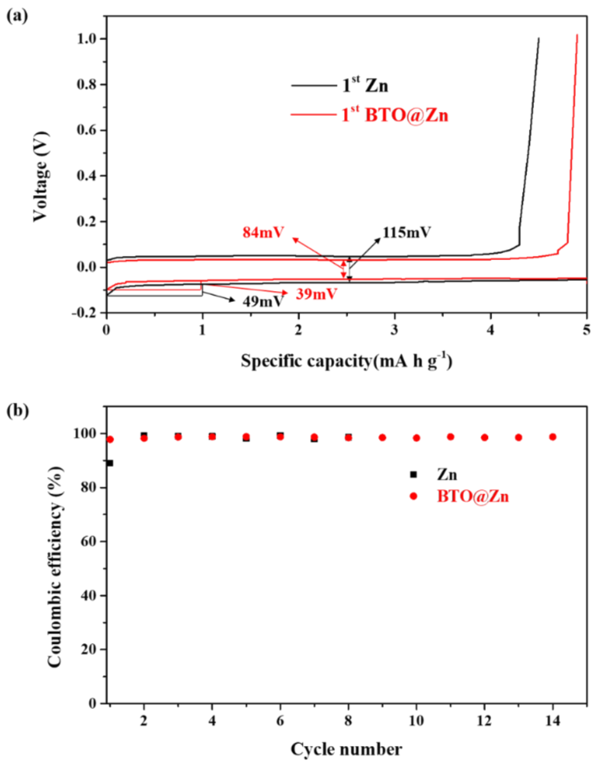

Fig. S7 (a) Voltage profiles of the initial cycle of Zn-Ti cells with bare Zn and BTO@Zn at 5 mA cm^{-2} with capacity of 5 mAh cm^{-2}. (b) The Coulombic efficiency of Zn-Ti cells with bare Zn and BTO@Zn at 5 mA cm^{-2} with capacity of 5 mAh cm^{-2}

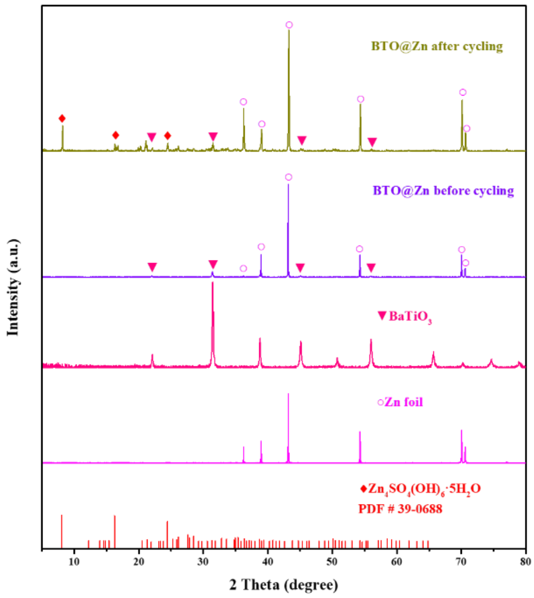

Fig. S8 XRD patterns of the BTO@Zn after 50 cycles at 1 mA cm^{-2} with areal capacity of 1 mAh cm^{-2}

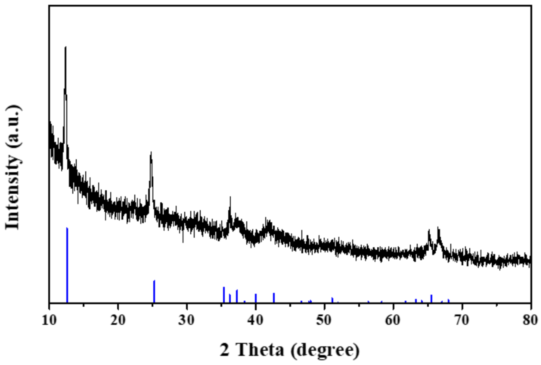

Fig. S9 XRD pattern of the MnO_2

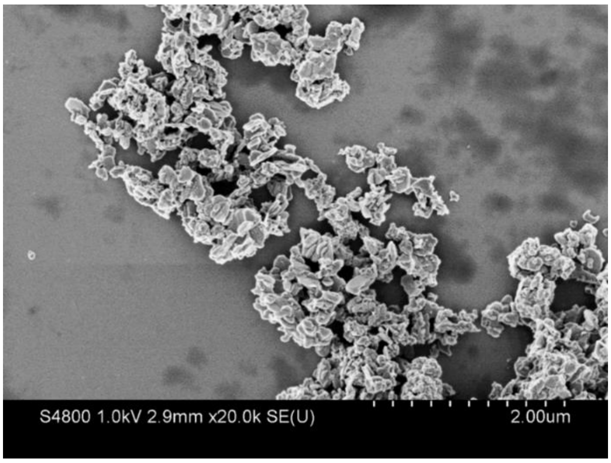

Fig. S10 SEM image of the MnO_2

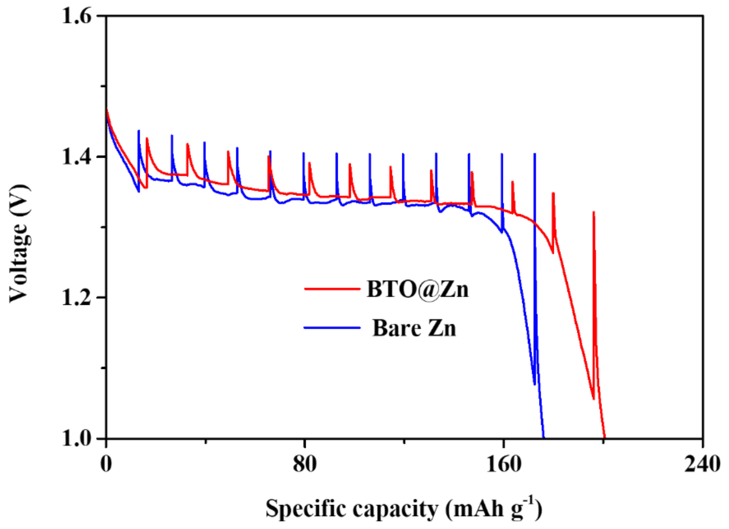

Fig. S11 The discharge GITT profiles for initial cycle of Zn-MnO_2 batteries based on BTO@Zn and bare Zn. The batteries were discharged at 0.05 A g^{-1} for 20 min, and took a rest for 120 min

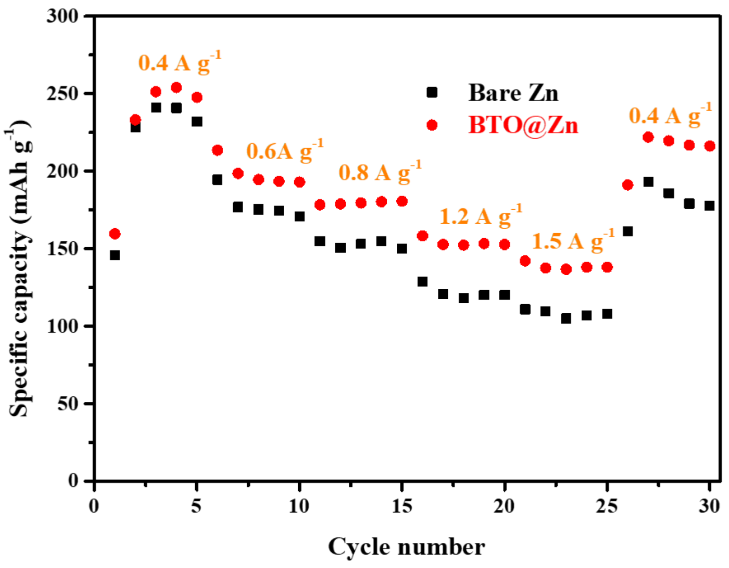

Fig. S12 The rate capability of Zn-MnO_2 batteries based on bare Zn and BTO@Zn
